# Small Interfering RNA (siRNA) as a Targeted Therapy for Acute Respiratory Distress Syndrome: Evidence from Experimental Models

**DOI:** 10.3390/ijms27020717

**Published:** 2026-01-10

**Authors:** Viktoriia Kiseleva, Polina Vishnyakova, Andrey Elchaninov, Ivan Kiselev, Gennady Sukhikh, Timur Fatkhudinov

**Affiliations:** 1 National Medical Research Center for Obstetrics, Gynecology and Perinatology Named after Academician V.I. Kulakov of Ministry of Healthcare of Russian Federation, 117198 Moscow, Russia; 2 Research Institute of Molecular and Cellular Medicine, Peoples’ Friendship University of Russia (RUDN University), 117198 Moscow, Russia; 3 Avtsyn Research Institute of Human Morphology of Federal State Budgetary Scientific Institution “Petrovsky National Research Centre of Surgery”, 117418 Moscow, Russia; 4 Chazov National Medical Research Center of Cardiology, Ministry of Health of the Russian Federation, 121552 Moscow, Russia

**Keywords:** ARDS, acute respiratory distress syndrome, siRNA, animal models, gene knockdown, preclinical research

## Abstract

Acute Respiratory Distress Syndrome (ARDS) is a severe complication of acute lung injury (ALI) characterized by acute hypoxemic respiratory failure and diffuse alveolar damage, with a high mortality rate and a current lack of treatments beyond supportive care. Its complex pathophysiology involves immune cell activation, pro-inflammatory cytokine release, and disruption of the alveolar–capillary barrier, leading to pulmonary edema and fibrosis. This review explores the potential of small interfering RNA (siRNA) therapy as a novel pathogenetic treatment for ARDS. The mechanism of RNA interference is described, highlighting its high specificity for silencing target genes. The paper then evaluates various animal models used in ARDS preclinical research, noting the advantages of large animals (pigs) for their physiological similarity to humans and the suitability of rodents for studying long-term fibrotic stages. Finally, the review summarizes promising in vivo studies where siRNA-mediated knockdown of several genes (e.g., *TIMP1*, *BTK*, *LCN2*, *HDAC7*, *CCL2*, *NOX4*, *TNFα* and *TLR4*) significantly reduced inflammation, improved lung histology, and increased survival. The collective evidence underscores siRNA’s considerable potential for developing targeted therapies against ARDS, moving beyond symptomatic care to address the root molecular mechanisms of the disease.

## 1. Introduction

Acute respiratory distress syndrome (ARDS) remains a leading cause of respiratory failure and high mortality in intensive care units [1]. It is characterized by poor oxygenation and non-compliant or “stiff” lungs. According to the Berlin definition, ARDS is defined by acute onset, bilateral pulmonary infiltrates on chest X-ray or computer tomography of non-cardiac origin, and a PaO_2_/FiO_2_ ratio of less than 300 mmHg [2]. The mortality rate associated with ARDS varies depending on the severity of the condition and the study population. Based on an analysis of 34 randomized controlled trials and 68 observational studies published between 2009 and 2019, Divyajot Sadana et al. showed that the weighted pooled mortality rate for ARDS was 39.4% (95% confidence interval, 37.0 to 41.8%) [3]. Modern guidelines for the treatment of ARDS include only symptomatic therapy, such as ventilator support, prone positioning, sedation with medications to prevent movement, diuretic medication for excess fluid removal, and extracorporeal membrane oxygenation (ECMO) [4].

The pathophysiology of ARDS involves activation of immune cells, release of pro-inflammatory cytokines, damage to the alveolar–capillary barrier, and apoptosis of lung cells [5]. ARDS is a polyetiological disease. The main risk factors can be divided into two groups: direct lung injury, which causes pulmonary ARDS (e.g., aspiration syndrome, inhalation of toxic substances, pulmonary infection, blunt chest trauma), and indirect lung injury, which causes extrapulmonary ARDS (e.g., shock, sepsis, trauma, blood loss, blood transfusions, poisoning, artificial circulation), where the triggering insult originates outside the lungs. These two etiologic subtypes result in different changes in mechanical ventilation, cellular response, and gene expression (Table 1) [6].

In pulmonary ARDS, direct damage to the bronchial and alveolar epithelium (due to infection, pulmonary contusion, drowning, etc.) results in bronchial obstruction, atelectasis, and alveolar edema [7]. In these patients, alveolar edema and fibrin accumulation in the alveoli predominate, and in later stages, large numbers of collagen fibers and apoptotic neutrophils are observed [7,8]. Single-cell sequencing has shown that pulmonary ARDS is characterized by significant levels of B cells, neutrophils, and Th cells, with low numbers of basophils, macrophages, monocytes, and dendritic cells (DCs) in lung tissue compared to extrapulmonary ARDS [9].

In extrapulmonary ARDS, damage to the endothelium of pulmonary capillaries leads to metabolic and structural changes. As a result, the permeability of the alveolar–capillary barrier increases. These changes are associated with the release of plasma and blood cells into the lung interstitium, resulting in significant thickening of the interalveolar septa. In this case, the pathological changes in the lungs are more diffuse, with a predominance of alveolar collapse compared to pulmonary ARDS [10].

**Table 1:** Key differences between pulmonary and extrapulmonary ARDS.

Pulmonary or Direct ARDS	Extrapulmonary or Indirect ARDS
Causes [1,6,11]	
aspiration syndrome, inhalation of toxic substances, pulmonary infection, blunt chest trauma, etc.	shock, sepsis, trauma, blood loss, blood transfusions, poisoning, artificial circulation, etc.
Lung structures damaged first [12]	
Alveolar epithelium	Pulmonary endothelium
Differences in pathogenesis [12]	
alveolar edema	interstitial edema
Changes in cell level [9]	
B cells, neutrophils, Th cells predominant	basophil, macrophage, monocyte and dendritic cells predominant
Changes in gene level [9]	
*Clec4e*, *Retnlg* and *S100a9*	*Coro1a* and *Lars2*
Prognosis [10,12]	
Low potential to alveolar recruitment	High potential to alveolar recruitment

The diffuse inflammation of the lungs in ARDS is divided into three phases: exudative, proliferative, and fibrotic [10,13]. In healthy lungs, endothelial and alveolar epithelial integrity is mediated by vascular endothelial cadherin (VE-cadherin) and E-cadherin, respectively. Type I and type II alveolar epithelial cells are involved in maintaining an osmotic gradient for the removal of fluid from the alveoli into the interstitium. A key component necessary for creating this osmotic gradient is the sodium channel (ENaC) [14,15]. Initially, the innate immune system is activated via Toll-like receptors on pulmonary epithelium and alveolar macrophages. Neutrophils are recruited to the lungs, and neutrophil extracellular traps (NETs) are formed with subsequent epithelial damage [16,17,18]. Thrombin, tumor necrosis factor-α (TNF-α), and vascular endothelial growth factor (VEGF) levels increase, leading to destabilization of VE-cadherin bonds. Simultaneously with the disruption of the alveolar–capillary barrier, alveolar fluid clearance is impaired, resulting in pulmonary edema [5,13]. Therefore, the first stage of the disease is called the exudative stage.

Resolution of inflammation requires restoration of the alveolar epithelial barrier, removal of inflammatory cells, and restoration of alveolar fluid clearance. Type II alveolar cells, fibroblasts, and myofibroblasts proliferate in this proliferative phase [13,19]. Disease development at this stage can follow two pathways [19]. The first leads to normalization of gas exchange through proliferation and differentiation of alveolar cells. The second—less favourable—pathway is associated with the penetration of fibroblasts into the alveolar spaces through gaps in the basal lamina [20]. Hyaline membranes are either removed by phagocytosis by macrophages or invaded by fibroblasts. In the interstitial space, which is filled with collagen and elastic fibres, interstitial fibroblasts (IFs) are located. A single-cell atlas of mouse and human fibroblasts in normal and diseased states, compiled by Matthew B. Buechler and colleagues, defines two major subpopulations of fibroblasts inhabiting different organs: universal and specialized (steady-state) or activated (perturbed-state) subtypes [21]. Close interaction between alveolar macrophages (AMs) and IFs is necessary for regulating extracellular matrix remodelling and the transition of fibroblasts into an active form with increased contractility (myofibroblasts) in both normal and pathological conditions [22,23].

The common complication of ARDS involves chronic fibrosis and occlusion of blood vessels [24,25]. Since ARDS is part of a systemic inflammatory response, the outcome of the pulmonary process depends on improvement of the systemic condition. The formation of interstitial and intra-alveolar fibrosis foci is associated with the proliferation of activated lung fibroblasts [26,27]. Their most important function is the synthesis of components of the extracellular matrix (collagen types I and III), which leads to slowly resolving fibrosis or irreversible changes in lung architecture. If the cause of ARDS is seasonal or avian influenza, the profile of molecules synthesized by fibroblasts may change. It has been shown that activated fibroblasts begin to produce A Disintegrin And Metalloproteinase with Thrombospondin Motifs 4 (ADAMTS4) protease, and high levels of its secretion are associated with severe disease and high patient mortality [27].

Existing therapy is predominantly supportive, which underscores the need to develop targeted treatments aimed at key pathways in the pathogenesis [28]. The discovery of RNA interference (RNAi) at the end of the 20th century by Andrew Fire and Craig Mello provided a powerful tool for regulating gene expression [29]. In this context, small non-coding RNAs, particularly Small interfering RNAs (siRNAs), represent promising candidates for the creation of new drugs for ARDS. The action of siRNA is based on the mechanism of RNA interference, which provides a biological response to double-stranded RNA (dsRNA) of exogenous and endogenous origin and regulates post-transcriptional gene expression.

## 2. The Mechanism of RNA Interference

RNAi is a highly conserved biological pathway for gene silencing. It functions as a vital defense mechanism against viruses and transposable elements [29]. The key effector molecules in this pathway are small non-coding RNAs, such as microRNAs (miRNAs), siRNA and piwi-interacting RNAs (piRNAs). siRNAs and miRNAs have similar gene silencing mechanisms. A detailed comparison of miRNA and siRNA as potential therapeutic agents is described in the papers by Lam JK et al. and Wang P et al. Therefore, below is Table 2 which reflects the main differences between these molecules [30,31,32].

The process of RNA interference can be broken down into four main stages: (1) initiation (dsRNA delivery/processing); (2) effector complex formation; (3) target recognition and cleavage; (4) amplification (in some systems) [29]. The initiation process begins with the presence of long, double-stranded RNA (dsRNA) in the cell. This can be introduced experimentally (for research or therapeutic purposes) or originate from viral replication, transgenes, or transposons. The RNase III enzyme Dicer recognizes and binds to this dsRNA. Dicer cleaves the dsRNA into short, double-stranded fragments approximately 21–23 nucleotides in length, with a characteristic structure: a 2-nucleotide overhang at the 3′ end of each strand, a 5′ phosphate group and a 3′ hydroxyl group. These fragments are now called small interfering RNAs. At the next stage the double-stranded siRNA is loaded into the heart of the RNAi machinery: the RNA-induced silencing complex (RISC). RISC is a multi-protein complex, with a member of the Argonaute (Ago) protein family (specifically Ago2 in mammals) serving as its catalytic core. The siRNA duplex is handed off from Dicer to RISC, often facilitated by chaperone proteins like Hsc70/Hsp90. Within RISC, the duplex is unwound in an ATP-dependent process. One strand, known as the passenger strand (or sense strand), is selected for degradation. The other strand, the guide strand (or antisense strand), is retained within the complex and bound by the Argonaute protein. This strand determines the target specificity of the activated RISC, now referred to as siRISC. After that the activated siRISC, now programmed with its guide strand, scans the cellular pool of mRNA in search of its target. The guide strand base-pairs with its complementary mRNA sequence with perfect or near-perfect complementarity. Once bound, the Ago2 protein, which possesses an RNase H-like domain called the PIWI domain, acts as a “Slicer” enzyme. Ago2 cleaves the target mRNA precisely between nucleotides 10 and 11 relative to the 5′ end of the siRNA guide strand. This cleavage produces two mRNA fragments: a 3′ fragment with a 5′ cap, which is rapidly degraded by exonucleases (e.g., XRN1) and a 5′ fragment with a poly-A tail, which is degraded by the cytoplasmic exosome complex. This catalytic cleavage event destroys the mRNA, preventing its translation into protein and thus achieving post-transcriptional gene silencing [37,38].

Under physiological conditions, siRNA in a cell is derived from long, double-stranded RNA of various endogenous (or exogenous) origins. Endogenous sources of siRNA in mammalian cells include (dsRNAs) from transposable elements and repetitive sequences, long hairpin RNAs (hpRNAs), and complementary sense and antisense transcripts that are co-expressed from the same or different loci [39]. Exogenous sources of siRNA in mammalian cells include synthetic siRNAs, transgenes, and viruses [40].

Numerous studies show that siRNA loading into RISC is a multi-step and heterogeneous process: thermodynamic asymmetry, protein sensors that interpret asymmetry (Dicer/R2D2/TRBP), Ago conformational changes, and both slicer-dependent and slicer-independent unwinding govern strand choice and stability [40,41,42,43]. The use of chemical modifications reduces the influence of these factors on the loading of siRNA into the RISC [44]. For example, in a recent study by Evgenii Kliuchnikov et al., which involved determining thermodynamic stability (Tm) and activity (IC_50_) in vitro for 15 pairs of siRNAs (unmodified and chemically modified) followed by computer modeling to analyze structural, dynamic, and energetic properties and machine learning to identify key parameters affecting siRNA activity, it was found that low stability and high flexibility at the g2 and g6 positions of the guide strand contribute to maintaining the efficacy of chemically modified siRNAs [44].

A significant barrier for exogenous dsRNAs is the negatively charged cell membrane, which they must cross to enter the cytoplasm and be processed by the Dicer enzyme. Thus, overcoming the barrier created by the charged cell membrane is one of the key challenges in using siRNA, both in research and clinical practice. To address this, researchers use lipid particles that can easily fuse with the cell membrane, such as milk-derived exosomes, cholesterol-enriched apolipoprotein nanoparticles, genetically engineered bacterial outer membrane vesicles, nanovesicles coated with genetically modified HEK293T-ACE2 cell membranes, and synthetic lipid nanoparticles (LNPs) [45,46,47,48]. On the other hand, lipid nanoparticles can influence cellular uptake, biodistribution, and immune recognition, either enhancing or mitigating immune responses [49].

Upon systemic administration, siRNAs can activate the immune response through several pathways, primarily involving innate immune sensors that recognize siRNA as foreign RNA molecules. Systemically delivered siRNAs are often internalized by immune cells such as dendritic cells, macrophages, and monocytes via endocytosis. Inside endosomes, siRNAs can be sensed by TLR7 and TLR8, which recognize single-stranded RNA motifs rich in uridines or GU sequences. This recognition triggers a signaling cascade via the MyD88 adaptor protein, leading to the activation of NF-κB and interferon regulatory factors (IRFs), which drive the production of type I interferons (IFN-α and IFN-β) and proinflammatory cytokines like TNF-α and IL-6 [50]. It has been shown that siRNA sequences that are U-rich or contain GU motifs tend to be more immunogenic because of their recognition by TLR7/8 [51].

If siRNAs escape the endosomes or are delivered directly to the cytoplasm, they may activate cytosolic RNA sensors like RIG-I (retinoic acid-inducible gene I) or MDA5 (melanoma differentiation-associated gene 5). These sensors detect dsRNA structures and initiate a signaling cascade that also results in type I interferon production and an antiviral state [51]. Another significant problem with systemic administration is that lipid nanoparticles are largely retained in the liver [52]. To minimize the activation of the innate immune system, when designing siRNA sequences, researchers avoid known immunostimulatory motifs and use chemical modifications such as 2′-O-Methyl (2′-OMe), 2′-Fluoro (2′-F), and 2′-Methoxyethyl (2′-MOE). These substitutions on the ribose sugar shield the siRNA from immune recognition, particularly by TLR7/8, and increase stability [53]. Phosphorothioate (PS) linkages, which replace a non-bridging oxygen with sulfur in the phosphate backbone, improve nuclease resistance and can alter protein binding, thereby reducing immunogenicity. High chemical purity also plays a crucial role in avoiding immune response activation [53].

In the context of using siRNA to treat acute respiratory distress syndrome, intranasal delivery is often employed. This approach also utilizes lipid nanoparticles with a specific set of ionizable cationic lipids, siRNAs conjugated with specific peptide motifs, inhalable polyplexes based on polyethylene glycol-poly(β-amino ester)-histidine (PEG-PBAE-His, PPH) that combine pH sensitivity, enhanced stability, and low toxicity, cationic liposomes (e.g., siPFKFB4/PRLPTX@RBCM-cRGD) that simultaneously deliver a drug and a siRNA, lipid nanoparticles formed by hybridizing a charge-inverting pH-dependent lipid film with apoptotic T-cell membranes, and mesoporous polydopamine nanoparticles (MPDA) coated with cationic polyethylenimine (PEI) and macrophage-targeting glucomannan (GM) (MPDA@PEI@GM NPs) [54,55,56,57,58,59].

Another example is a biomimetic drug delivery system, TP-siRC@tHyNPs, created by fusing exosomes derived from engineered cells overexpressing DR5 single-chain variable fragments (DR5-Exo) with liposomes co-encapsulating triptolide (TP) and CYP3A4-siRNA [60].

The use of viral vectors as a tool for siRNA delivery is also noteworthy. Retroviruses and lentiviruses are known for stable genome integration, enabling long-term expression but carrying risks of oncogenesis. Adenoviruses and AAVs are used for transient expression, often with cell type-specific targeting and lower integration risks. HSV vectors, notably used in neurological and oncolytic applications, have been primarily investigated in vitro for RNAi delivery. Emerging self-replicating cytoplasmic RNA viruses like VSV show promise for high expression and oncolytic cancer therapy. For example, adeno-associated virus serotype 6 (AAV6) has been successfully used as a viral gene vector to transduce airway epithelial cells and reduce mucin 5AC (*MUC5AC*) expression for the treatment of asthma [61]. Despite the variety of approaches to targeted delivery of siRNA and approaches to increasing stability, today there is no standardized protocol for siRNA production, its modifications and delivery to target cells.

## 3. Animal Models of ARDS for Preclinical Studies

For the reproduction of ARDS in animals as close as possible to humans, the appearance of the following pathological processes is important: a neutrophilic alveolitis, deposition of hyaline membranes and formation of microthrombi [19]. Based on the degree of lung damage, approaches to creating a model can be divided into three large groups: direct, indirect and combination lung damage [62]. The first group includes intranasal or intratracheal administration of damaging components: bacteria or bacterial products, acids, gastric contents, etc. Indirect lung damage is imitated by the development of an acute systemic inflammatory reaction (sepsis, trauma, coronary artery bypass grafting, gastric contents abandonment). The third group, according to the name, includes combinations of the first and second groups [62]. 

Choosing relevant animals for the ARDS model remains a non-trivial task. One should take into account not only practical factors like the animal size and availability of species-specific reagents, which improve a model’s usability, but also biological characteristics, such as the functional specificities of the immune system, that affect its validity. In the context of preclinical research, the most interesting works are those that reproduce ARDS in large animals with manifestations closest to human ones. Pigs are of the greatest interest, as they have an anatomical structure similar to humans [63,64].

In the work of Evan P. Rotar et al., ARDS were reproduced on Yorkshire pigs with an average weight of 55.5 kg [64]. The study included 12 animals, of which 4 were control animals. A combined “two-stroke” model based on aspiration of gastric contents was used (administration of 3 mL/kg of gastric secretions (pH 1.2) into both main bronchi under bronchoscopic control) and ventilator-associated lung injury. After 12 h, the animals were removed from the experiment and histological changes in the lungs and changes at the systemic level were evaluated. Locally, alveolar edema, neutrophil infiltration (PMN), the presence of hyaline membranes, and the development of interstitial inflammation were noted in the lungs. At the same time, there was a significant decrease in PaO_2_/FiO_2_ (<100 mmHg by 12 o’clock), the development of pulmonary edema and a decrease in lung compliance. The bronchoalveolar lavage fluid showed an increase in IL-6 and IL-8 in the lavage and lung tissue. The main advantage of the method is the combination of two factors (aspiration + ventilation), simulating a clinical scenario. However, this model is limited to reproducing only one etiology of ARDS and has a high mortality rate. Nevertheless, the model is suitable for preclinical trials of treatments aimed at restoring lung function, but requires caution due to the high mortality rate.

Kaslow S.R. et al. used a “two-stroke” model on 9 swine with an average weight of 49 kg: aspiration of gastric contents (bronchoscopic administration of standardized gastric secretions (pH 2.0) into both main bronchi (30–50 mL)) and systemic inflammation (intravenous infusion of lipopolysaccharide (LPS) *Escherichia coli* O55:B5 for 30–60 min) [64]. After a decrease in the PaO_2_/FiO_2_ ratio < 150 mmHg (“ARDS 0 h” moment), the animals were transferred to standard ARDS therapy. The experiment lasted 48 h. Interstitial and alveolar edema, neutrophil infiltration of the alveoli and respiratory tract, damage to the alveolar–capillary barrier (decreased expression of markers of epithelial (EpCAM, ZO3, pro-SPC) and endothelial (CD31, ZO1, P-selectin) cells) were noted in the lungs. At the systemic level, markers of inflammation in the blood serum (proinflammatory cytokines IL-1a, IL-6, IL-8, TNF-α, etc.) and acute phase proteins (D-dimer, CRP) increased. Leukopenia and thrombocytopenia were also noted. The resulting model has high clinical relevance, as it reproduces the key signs of ARDS in humans, provides the possibility of using standard therapy (ventilators, ECMO, vasopressors) and combines epithelial and endothelial damage. It allows us to study the pathogenesis and test new treatment methods in conditions as close as possible to clinical ones, but it requires significant resources and has limitations on the duration and reproducibility of histological features. And also, as in previous studies, it has a high mortality rate (3 out of 9 animals died before the planned end of the experiment) [64].

Russo C. et al. also used a two-factor model [65]. In large Yorkshire pigs weighing 59–71 kg, repeated bronchoalveolar lavages (BALs) were first performed using Triton X-100 solution to remove surfactant and create alveolar damage, then oleic acid (OA) mixed with autologous blood and saline solution was injected intravenously, which damaged the vascular endothelium. The administration of BAL and OA took place in several stages with an interval of about an hour. In the lungs, there was edema of the alveoli with pronounced infiltration of neutrophils and erythrocytes, pronounced thickening and replacement of the alveolar septa with hyaline membranes with fibrin deposits both in the alveoli and in small vessels. At the systemic level, pronounced hypoxemia was noted (the PaO_2_/FiO_2_ ratio dropped to 76.8 mmHg), a significant decrease in pulmonary compliance (lung elasticity) by an average of 65%, correlated with the severity of damage and the development of acidosis with a progressive decrease in pH and an increase in partial pressure of CO_2_. An increase in the concentration of pro-inflammatory cytokines, such as IL-1b, IL-6, IL-8, was noted, especially after administration of oleic acid. Thus, among the advantages of the model, it is worth noting the reproduction of key pathophysiological stages of human ARDS (exudative, proliferative and fibrotic phases), the possibility of achieving a stable ARDS state with severe hypoxemia and decreased pulmonary compliance, as well as maintaining long-term monitoring and assessment of disease dynamics, including changes in gas exchange, hemodynamics and inflammatory markers. Among the disadvantages are the possibility of sudden death of animals due to severe cardiorespiratory complications, for example, massive pulmonary embolism, the complexity of the procedure with the need for multiple measures (several bronchoalveolar lavages and subsequent OA infusions). Thus, the two-factor model with BAL and OA in pigs provides a realistic and reproducible ARDS condition close to human clinical, physiological and histological criteria, which makes it promising for research into new therapeutic approaches.

In the Tiba M.H. et al. study, also conducted on 14 Yorkshire-mix pigs, the ARDS model was reproduced by combining the effects of two types of lung damage: systemic—by injecting live cultures of *Escherichia coli* directly into the kidney parenchyma to provoke sepsis and systemic and local inflammation (volutrauma (ventilation with high respiratory volume (12–15 mL/kg)) or hyperoxia (breathing 100% oxygen) or aspiration of gastric contents (bronchoscopic administration of acidic gastric particles (pH = 1) into the pulmonary bronchi)) [66]. Only in animals with combined exposure, significant diffuse alveolar damage (DAP) was detected—hyaline membranes, intra-alveolar edema, fibrin thrombi, which corresponds to the histopathological criterion of ARDS in humans. There was a significant deterioration in oxygenation: PaO_2_/FiO_2_ decreased by the 12th hour (severe ARDS) and the appearance of bilateral diffuse darkening in the lungs, meeting the criteria of ARDS according to the Berlin definition, was noted. Thus, the authors have developed a highly accurate pig model of ARDS, which reliably reproduces the key pathophysiological, radiological and histological signs of the disease in humans. The model is based on the combined effects of direct and indirect lung injury, which corresponds to the multicomponent nature of ARDS in patients in clinical practice.

Leiphrakpam P.D. et al. modeled ARDS in 21 female pigs by inhaling oak smoke through an endotracheal tube for 2 h, preventing the development of accompanying skin burns, a unique feature of this model [67]. After 48 h, the animals were sacrificed. Alveolar edema, intra-alveolar hemorrhages, leukocyte infiltration, and an increase in total protein in the BAL fluid were observed (*p* = 0.0436). Systemic changes included hypoxemia (a decrease in SpO_2_ by ~31%, PaO_2_/FiO_2_ by ~208 (50%)), hypercapnia (an increase in PaCO_2_ and ETCO_2_), acidosis, decreased saturation (SaO_2_ and SmvO_2_ decreased by 39–43%), decreased blood oxygen content: CaO_2_ and CmvO_2_, and bilateral infiltrates on radiographs. A key advantage of this model was the extended post-injury observation period (48 h), which had not been achieved in previous studies. The developed porcine model with isolated smoke inhalation is a robust and reproducible model for studying ARDS induced by toxic inhalation. It reproduces key pathophysiological and clinical features of the disease and can be used for preclinical evaluation of new therapeutic strategies. However, the model requires significant resources and optimization to reduce mortality and standardize smoke exposure.

In summary, porcine models of ARDS are highly relevant for preclinical studies due to their pathophysiological proximity to the human pathology. It was particularly demonstrated in a study investigating total liquid ventilation (TLV) using a new liquid ventilation for breathing (LV4B) prototype in pigs with severe ARDS induced by oleic acid [68].

Although pigs as animals anatomically similar to humans offer significant advantages, porcine models of ARDS are universally characterized by a major limitation: rapid animal mortality. Thus research on smaller species remains the most accessible approach for studying ARDS and testing potential therapeutics in long-term follow-up studies, which are currently unattainable with larger animal models. Furthermore, while protocols for modeling ARDS in pigs are trending toward standardization (e.g., the common use of two-hit injury models), the methods for replicating ARDS in smaller animals are highly diverse and thus offer more possibilities in studying specific ARDS subtypes.

The relative ease of handling and proven similarity between the pharmacokinetics of some antibacterial drugs in rabbits and humans support the use of these large rodents for ARDS modelling in translational studies. A body of research using rabbit ARDS models worth mentioning.

Gras E. et al. and Nguyen N.T.Q. et al. in their studies used endobronchial administration of a highly virulent and antibiotic-sensitive *Pseudomonas aeruginosa* strain (Pa6206), which produces the exotoxin ExoU, to rabbits (New Zealand White) [69,70]. To demonstrate that symptoms were caused by a live infection, rather than simply an inflammatory response to bacterial components, the control group was injected with UV-killed Pa6206 or Ringer’s solution. To simulate non-ventilated pneumonia, in the first study, the endobronchial tube was immediately removed after bacterial administration, while in the second study, artificial ventilation (IVL) with a low tidal volume was used. In the first case, animals were sacrificed after 23 h; however, animals showing signs of respiratory failure were euthanized 13 h after infection. Hypoxemia (PaO_2_ < 60 mmHg) and the first histological signs of pneumonia were observed as early as 5 h after infection. However, no such changes were observed in the control group (killed bacteria) [69]. In the second case animals were withdrawn from the experiment after 48 h [70]. Histological changes characteristic of ARDS were observed in the lungs (edema, exudate/fibrin, PMN, an increase in the lung weight to body weight (LW/BW) ratio, which is a marker of pulmonary edema) and changes at the systemic level (hypoxemia (progressive decrease in PaO_2_:FiO_2_ to a level of <100, characteristic of severe ARDS), leukopenia, neutropenia, thrombocytopenia, hyperlactatemia (lactate > 10 mmol/L), acidosis, hypotension, bacterial dissemination from the lungs to the heart, liver, spleen, kidneys, and multiple organ dysfunction, manifested by an increase in markers of damage to the heart (troponin I, CK-MB), liver (ALT, AST), kidneys (creatinine, urea)). Despite the limitations of the method—the use of only one strain (sensitive to antibiotics), complications of mechanical ventilation such as endotracheal tube obstruction with mucus (resulting in the death of three rabbits), and high resource requirements due to the need for 24 h animal monitoring and a large team of specialists—the model reproduces the key features of ARDS in humans, making it highly clinically relevant, and low-tidal volume mechanical ventilation simulates clinical conditions. The model also allows for testing antibiotics and intensive care unit (ICU) therapies (norepinephrine, infusion support) while utilizing comprehensive monitoring (hemodynamics, blood gas composition, etc.) of the animals’ condition. It reproduces the key pathophysiological and clinical features of the disease, including multiple organ dysfunction and septic shock. However, the model requires further refinement to study resistant strains and delay treatment initiation. Despite the differences in ARDS modeling in these animals, human-like pharmacokinetics of drugs (such as meropinem) have been demonstrated, demonstrating the importance of such models in preclinical studies [69,70].

Petra Košútová et al. demonstrated the relevance of another rabbit model of acute respiratory distress syndrome (ARDS), induced by hydrochloric acid aspiration and mechanical ventilation-induced lung injury, for the preclinical evaluation of new therapeutics [71]. While the two previous studies were narrowly focused on identifying drugs targeting a single bacterial species, this model appears more versatile. The study, also conducted in rabbits, combined HCl aspiration with subsequent aggressive mechanical ventilation. The authors demonstrated that the model reliably recreates key pathophysiological features of severe ARDS, including impaired gas exchange, severe inflammation, oxidative stress, edema, and structural lung damage [71].

**Figure 1:**
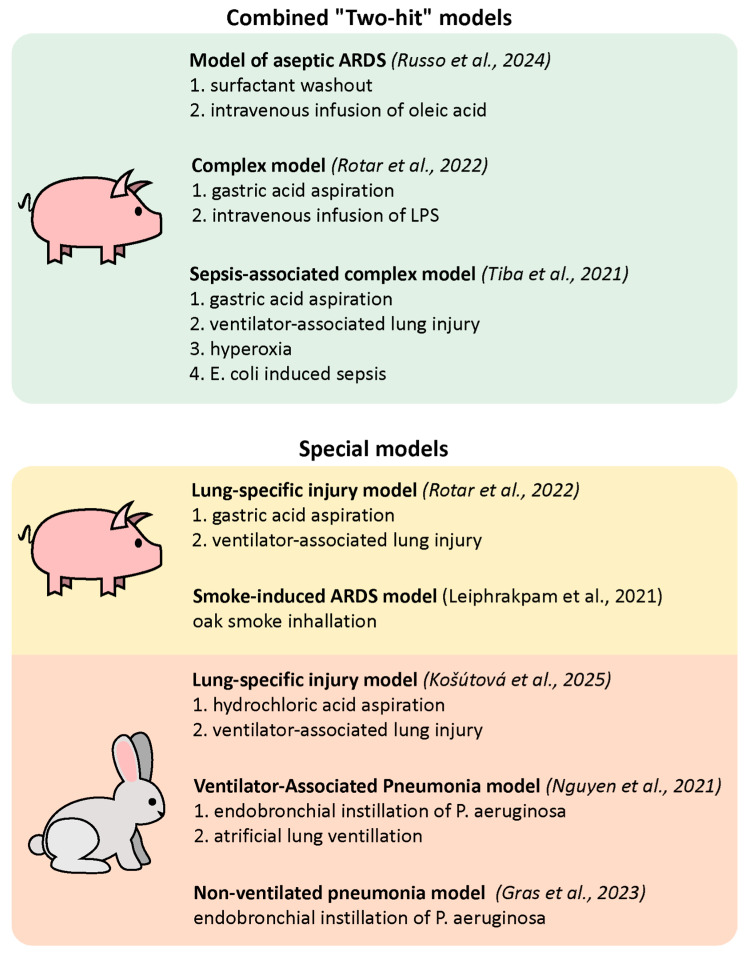
Clinically relevant models of ARDS in large animals. Russo et al., 2024 [65], Rotar et al., 2022 [63], Tiba et al., 2021 [66], Leiphrakpam et al., 2021 [67], Košútová et al., 2025 [71], Nguyen et al., 2021 [70], Gras et al., 2023 [69].

Figure 1 summarizes data on clinically relevant large animal models of ARDS discussed above.

Despite all the advantages of large animals, small rodents—mice and rats—remain the animals of choice for studying ARDS pathogenesis and searching for novel therapeutic approaches in basic research. The specifics of reproducing ARDS in small rodents have been described in detail in the works of Aeffner F. et al. and Chu K.A. et al. [72,73]. In this paper, we would like to highlight the only new experimental work in this area.

A recently published study by Chu K.A. et al. male Sprague-Dawley rats were given a single intratracheal injection of bleomycin (BLM) followed by restraint in a left lateral decubitus position at a 60-degree angle for 90 min to ensure preferential bleomycin action in the left lung, minimizing damage to the right lung and allowing the animal to survive [72]. A second key point was that animals were observed and assessed for 7 days after injury induction. Histologically, progressive and severe changes were noted in the left lung. On day 1 researchers noted the beginning of cellular infiltration into the interstitium and alveoli. During days 2–7, progressive cellular infiltration led to the widespread degradation of alveolar architecture, which was nearly complete in the central lung regions. Identifiable alveoli were confined to the parenchymal periphery. By day 7, the volume of the left lung had significantly decreased (from ~280 mm^3^ to ~195 mm^3^), and the airspace had narrowed. The area occupied by cellular infiltrate and compacted tissue progressively increased, reaching 68.2% of the left lung volume by day 7. Sirius red staining showed a significant increase in collagen deposition (fibrosis) by day 4 (30.3%) and day 7 (25.5%) compared to the control group (13.4%). At the systemic level, the development of severe respiratory failure and a systemic inflammatory response were noted: a sharp deterioration in oxygenation parameters: arterial oxygen saturation (SpO_2_) decreased from 97.9% to 83.7%, and the partial pressure of oxygen in arterial blood (PaO_2_) fell from 89.2 mmHg. to 65.3 mmHg by day 7. Partial pressure of carbon dioxide (PaCO_2_) increased to 49.2 mmHg, indicating hypoventilation. Respiratory rate increased significantly from ~130 to ~329 cycles per minute, indicating severe dyspnea. Serum levels of proinflammatory cytokines and chemokines significantly increased: IL-5, IL-6, IFN-γ, MCP-1, MIP-2, G-CSF, and TNF-α. The animals experienced weight loss, decreased food intake, decreased muscle mass (decreased total protein in the gastrocnemius muscle), and a decrease in fat and lean mass below the diaphragm, as determined by micro-CT. Thus, on the one hand, the model reproduces the key features of clinical ARDS according to the Berlin definition: severe hypoxemia (PaO_2_/FiO_2_ < 100, with FiO_2_ set to 0.21), bilateral (radiographic) changes (although the injury is primarily unilateral, the systemic response is bilateral), and the absence of signs of heart failure, ensuring its clinical relevance. On the other hand, the model is limited by the use of bleomycin, a classic fibrosis inducer; in essence, it imitates the early fibroproliferative phase of ARDS.

Although this review section is devoted to in vivo models of ARDS, we would like to highlight the interesting work of Kewalramani N. et al. on the use of human precision lung sections (PCLS) for ex vivo reproduction of ARDS [74]. Such a model is a powerful addition to in vivo studies, not only validating their results on human tissues but also enabling deeper insights into molecular processes observed in model organisms. Increased levels of free heme in the lungs reported in in vivo studies were hypothesized to be one of the direct factors of lung injury manifested by inflammation and tissue damage [75]. However it could not be easily proved because the in vivo systems are too complex and the observed injury could be a secondary effect of other inflammatory mediators. The PCLS model directly tests this hypothesis. By applying a controlled dose of heme to human lung tissue and observing the direct effects—cell death, membrane damage, and specific pathway activation (*NF-κB*, *JAK/STAT*)—the study confirmed that heme is indeed a sufficient and direct trigger for key pathological events in ARDS. These data advanced the hypothesis from correlation (observed in vivo) to causation (proven ex vivo). The PCLS model, as the authors note, is excellent for screening. Researchers can quickly test multiple drugs, siRNAs, or inhibitors (such as an NF-κB inhibitor) on tissue sections. Positive results obtained ex vivo can then be prioritized for further testing in complex and expensive in vivo models, greatly increasing the efficiency of the overall research process. However, the authors acknowledge that PCLS is an isolated system and does not take into account the impact of surrounding cells on the pathogenesis of ARDS [74].

In general, the choice of the model and method of reproducing ARDS depends on the goals of the experiment and the stage of the disease being studied. For example, pigs or rabbits are optimal to study early stages of ARDS, since they can quickly and reliably reproduce the key histopathological and physiological signs of the human exudative phase: neutrophilic inflammation, alveolar edema, hyaline membrane formation and severe hypoxemia [63,64,65,66,67,68,69,70,71]. On the other hand, the study of the proliferative and fibrotic phase, characterized by the onset of tissue repair, proliferation of fibroblasts and early collagen deposition, requires long-term observation of the animal’s condition. The best animals to study this stage of the disease are small rodents mostly due to their superior survival rates [73].

The correct choice of model is also important for assessing the effectiveness of siRNA therapy. Since siRNA is designed to suppress a specific gene involved in the development of a disease, the model must reproduce the disease-associated molecular mechanisms in which the target gene is active. Obviously, using the wrong model may lead to a false negative result. For example, antifibrotic siRNAs most likely will not show significant effects in ta short-term porcine models of ARDS. Conversely, interspecies divergence in immune system biology may lead to the limited efficacy of antineutrophil siRNAs in mice and rats [76].

## 4. In Vivo Studies Using siRNA to Treat ARDS

siRNA therapy has great potential in the field of pathophysiological treatment of acute respiratory distress syndrome. Despite the fact that to date, two drugs ONPATTRO^®^ (patisiran) (Alnylam Pharmaceuticals, Inc., Cambridge, MA, USA) have been approved for clinical use (used to treat polyneuropathy in hereditary transthyretin-mediated amyloidosis) and GIVLAARI^TM^ (givosiran) (Alnylam Pharmaceuticals, Inc., Cambridge, MA, USA) (treatment of acute hepatic porphyria (AKI)), there are no registered clinical studies of drugs for the treatment of ARDS based on siRNA [77,78]. However, there are a number of studies in animal models that show not only the role of the studied gene in the pathogenesis of ARDS, but also a potential treatment using siRNA (Table 3 and Table 4). The efficacy of siRNA therapy was assessed using histological, biochemical, and functional parameters. The target genes in the presented works were:

*BTK* encodes Bruton’s tyrosine kinase which is widely expressed in myeloid cells and regulates ROS production, cytokine release, and survival in macrophages and neutrophils, thus shaping systemic inflammatory responses [79,80]. Bruton’s tyrosine kinase integrates TLR and Fc-receptor signaling in multiple settings, positioning it as a nodal kinase for innate immune overactivation seen in sepsis-associated ARDS [79,81]. The key role of *BTK* in the development of ARDS against the background of COVID-19 was shown in the treatment of 19 patients with severe COVID-19 with a selective inhibitor—Acalabrutinib [82].

*CCL2* encodes C-C Motif Chemokine Ligand 2, also known as MCP-1, which is a crucial signaling protein (cytokine) that acts as a “chemical beacon,” powerfully attracting immune cells like monocytes and macrophages to sites of inflammation, infection, or injury, playing vital roles in immunity, tissue repair, and disease (like cancer, fibrosis, and neurological disorders) by controlling immune cell traffic and influencing cellular processes [83]. *CCL2* is consistently upregulated in experimental and human ARDS and drives monocyte/macrophage and neutrophil recruitment [84,85].

LCN2 encodes Lipocalin-2 or Neutrophil gelatinase-associated lipocalin (NGAL) which is a circulating adipocytokine essential for the transport of small and hydrophobic molecules (steroids, free fatty acids, prostaglandins, and hormones) to target organs. *LCN2* expression has been shown to be increased in acute lung injury and often exacerbates inflammation, oxidative stress, and ferroptosis [86,87].

*TIMP1* encodes Tissue inhibitor of metalloproteinases-1 which is regulates matrix turnover and also has cytokine-like functions. In ARDS/ALI, it is both a biomarker of severity and an active modulator of inflammation, extracellular matrix (ECM) remodeling, and vascular permeability [88,89].

*HDAC7* encodes Histone deacetylase 7 (HDAC7) which is a class IIa zinc-dependent enzyme that regulates gene expression by removing acetyl groups from histones. It’s involved in immune regulation, endothelial biology, and fibrosis. In experimental lung injury, it emerges as a key amplifier of Gram-negative pneumonia–induced ALI, the main pathological substrate of ARDS [90,91].

*TNF-α* encodes Tumor Necrosis Factor alpha (TNF-α) which is a powerful pro-inflammatory cytokine, a chemical messenger from the immune system, produced mainly by macrophages, that regulates inflammation, cell death (apoptosis), fever, and the body’s response to infection and cancer [92]. While essential for normal functions like neurogenesis and tissue repair, overproduction of TNF-α is a key driver in autoimmune diseases (like rheumatoid arthritis and Crohn’s) and other conditions, leading to the development of anti-TNF therapies that block its action. TNF-α is a central driver of ARDS pathophysiology, promoting inflammation, endothelial and epithelial barrier failure, and impaired alveolar fluid clearance, and its levels track with ARDS risk and poor outcomes. However, TNF-α also contains a lectin-like domain and engages TNFR2 pathways that protect lung barrier and fluid transport, so future therapies are focusing on receptor- and domain-selective modulation rather than global TNF blockade [93].

*NOX4* encodes NADPH Oxidase 4 which is a crucial enzyme that generates ROS, primarily hydrogen peroxide, within cells, acting as an oxygen sensor and influencing various cellular processes like growth, differentiation, and signaling, particularly in the kidney, heart, and brain, with its activity linked to aging, cancer, and conditions like fibrosis and heart failure. Unlike other NOX enzymes, NOX4 is constitutively active, meaning it doesn’t require complex activation, and is localized to the endoplasmic reticulum, mitochondria, and nucleus, interacting with p22phox for function [94].

*TLR4* encodes Toll-like receptor 4 which is a crucial transmembrane protein in the innate immune system, acting as a primary sensor for bacterial components like LPS from Gram-negative bacteria, as well as endogenous molecules from damaged tissues, initiating strong inflammatory responses by activating intracellular signaling pathways (MyD88/TRIF) that lead to pro-inflammatory cytokine production. It’s vital for fighting infections but also implicated in chronic inflammatory diseases and aging, making it a significant therapeutic target for conditions like sepsis, autoimmune disorders, and age-related condition [95].

For example, in the work of Ivan V. Chernikov et al., they showed that suppression of the expression of the *TIMP1* involved in the processes of matrix reorganization leads to a decrease in the number of neutrophils in bronchoalveolar lavage and a decrease in the thickness of the interalveolar septa to close to physiological values [96]. It is worth noting that in this work, a single intranasal siRNA injection was rather preventive in nature, since it was carried out 8 days before the induction of ALI by LPS in the mouse model. The data obtained indicate that anti-*Timp1*-siRNA reduces the severity of LPS-induced acute lung injury. Liposomal delivery system based on polycationic amphiphile 1,26-bis (cholest-5-en-3-yloxycarbonylamino)-7,11,16,20-tetraazagehexacosane tetrahydrochloride (2X3) and lipid-helper dioleoylphosphatidylethanolamine (DOPE) was used for siRNA delivery [96].

A significant potential for use as a therapeutic agent is the *BTK* encoding an enzyme essential for B cell development, function, and signaling in the immune system. Suppression of its expression was carried out by the teams Zhou et al. vs. Krupa et al. on ARDS models in mice reproduced in various ways [97,98].

In the first case, the cecal ligation and puncture (CLP) approach was used, simulating intestinal perforation and polymicrobial peritonitis. As in the case of suppression of *Timp1* expression, the therapeutic drug was used before the reproduction of ARDS, namely one hour before the operation [96]. The injection was performed intratracheal, without the use of additional components for the targeted entry of siRNA into the lung tissue.

In the study of Krupa A. et al., the authors used a unique “two-hit” model of LPS/immune complexes (IC)-induced ARDS to model the disease, which, in their opinion, more accurately reflects the clinical situation in patients [97]. The first “shock” or priming consisted of intraperitoneally injecting LPS. After 8 h, a second “kick” or challenge was performed—mice were intranasally injected with immune complexes (IC), which are antibodies against chemokine KC (CXCL1) and chemokine KC itself (anti-KC:KC ICs). These complexes mimic pathogenic immune complexes detected in pulmonary edema fluid in patients with ARDS. The authors showed that such a combined model leads to severe lung inflammation characterized by alveolar hemorrhage, thickening of the interstitium, the presence of alveolar exudate and infiltration of inflammatory cells (neutrophils). The siRNA construct siRNA + F(ab’)_2_ fragments of antibodies against neutrophils (antibody Ly-6G1A8, which binds to the surface marker of neutrophils Gr-1) was used to deliver siRNA to neutrophils. Conjugated siRNA was administered intranasally after LPS pretreatment (after 8 h), but before the introduction of immune complexes (ICs).

Despite such significant differences in ARDS modeling and siRNA delivery to the *BTK*, in both studies, knockdown led to a significant protective effect and reduction in lung damage, which indicates a significant potential for using siRNA against the *BTK* as a pathogenetic therapeutic drug against ARDS.

Adipokine Lipocalin 2 (LCN2), also known as siderocalin, neutrophil gelatinase-associated lipocalin (NGAL), and uterocalin, has also been studied in the context of the pathogenetic therapy of ARDS [99]. A model of intraperitoneal administration of LPS was used to evaluate the effect of *LCN2* knockdown on the course of ARDS. A feature of this work was the systemic administration of plasmid with shRNA 1 h after injection of LPS. The knockdown of the *LCN2* led to a decrease in pulmonary edema, an improvement in the histological picture (a decrease in neutrophils, a decrease in hemorrhages, a decrease in edema of the alveolar walls, preservation of the structure of the alveolar sacs), a decrease in serum levels of proinflammatory cytokines (TNF-α, IL-1b, IL-8, MCP-1), a decrease in oxidative stress (a decrease in the level of malonic dialdehyde (MDA), a product of lipid peroxidation and increased activity of antioxidant enzymes: superoxide dismutase (SOD), glutathione (GSH) and catalase (CAT), inhibition of apoptosis, as well as inhibiting ferroptosis (knockdown *LCN2* suppressed key markers of ferroptosis, reduced the level of Fe^2+^ (iron ions) in lung tissue and the level of transferrin (Tf), a protein involved in iron transport, while increasing the level of proteins FTH1 (heavy chain ferritin 1) and GPX4 (glutathione peroxidase 4), which inhibits ferroptosis).

The use of *HDAC7* knockdown, whose main function is to suppress gene transcription by compacting chromatin, was proposed by a group of researchers from the USA [14,91,100]. It has been shown that inhibition of histone diacetylases leads to a decrease in the activity of heat shock protein-90 (HSP90), which is necessary for activation of the NF-kB pathway and triggering inflammatory reactions. Using an ARDS model reproduced by endotracheal administration of 30 × 10^6^ colony-forming units (CFU) of the Gram-negative bacterium *Escherichia coli* (serotype 06:K2:H1, strain 19,138), the effect of siRNA on the *HDAC7* was evaluated. siRNA was administered endotracheally 72 h before the introduction of bacteria and animals were removed 6 h after ARDS modeling. There was a decrease in inflammation (a decrease in the number of neutrophils in BAL and lung tissue), a decrease in edema and extravasation of red blood cells, a decrease in TNF-α levels in BAL, and an improvement in survival.

The siCCL2 assessment for ARDS in COVID-19 was conducted by Athaydes Seabra Ferreira H et al. two days before infection, K18-hACE2 transgenic mice were intravenously injected with hybrid polymer-lipid nanoparticles (NP) consisting of the cationic lipid-polymer compound 7C1 and PEG-lipid (C18-PEG5000) containing siCCL2 [101]. After that, a lethal dose (6 × 10^4^ BOE/mouse) of the Gamma (P.1) SARS-CoV-2 variant was administered intranasally and NP-siCCL2 was administered intravenously 24 and 72 h after infection. The knockdown of the *CCL2* led to significant positive changes in the lungs: a significant decrease in the recruitment of innate immune cells to the lungs, primarily monocytes and neutrophils, a decrease in the expression levels of the genes of proinflammatory cytokines IL-6 and IFN-γ, histologically in the group after treatment, there was a decrease in inflammatory infiltration, thickening of the alveolar walls, and general damage to lung tissue compared with infected, untreated mice. Unexpectedly, treatment with NP-siCCL2 resulted in a significant decrease in the number of viral particles (according to plaque formation and RT-PCR) in the lungs. The authors suggest that this is due to a decrease in the number of infiltrating macrophages and neutrophils, which can serve as a reservoir for the virus. In the spleen, treatment prevented an infection-induced decrease in the number of T-lymphocytes (CD3^+^), which indicates a decrease in systemic lymphopenia.

**Table 3:** Summary table of siRNA used in in vivo to treat ALI/ARDS.

Target	ARDS Model	Timing of siRNA Injection	Dose of siRNA	Knockdown Efficiency
Intranasal				
*TIMP1* (Tissue Inhibitor of Metalloproteinase 1) [96]	intranasal instillation of LPS (055:B5)	8 days before LPS introduction	2.5 µg siRNA per animal	Knockdown efficiency in vivo ~25%
*BTK* (Bruton’s tyrosine kinase) [97]	“two-hit” model of LPS/immune complexes (IC)-induced ARDS	8 h after LPS exposure but before anti-KC:KC IC administration. The authors believe this intervention will prevent further activation of alveolar neutrophils.	The authors do not indicate the dose of siRNA used.	The knockdown efficiency was 92%.
*CCL2* (CC Motif Chemokine Ligand 2) also known as MCP-1 [101]	K18-hACE2 transgenic mice with intranasal administration of a lethal dose (6 × 10^4^ PFU) of the Gamma (P.1) variant of SARS-CoV-2	2 days before SARS-CoV-2 infection And 1 day and 3 days post-infection (DPI)	1 mg/kg of body weight	50% knockdown of the infection-induced CCL2 mRNA upregulation
Intratracheal				
*BTK* (Bruton’s tyrosine kinase) [98]	cecal ligation and puncture (CLP)	1 h before CLP	4.2 mg/kg of body weight	The quantitative significance of the knockdown is not specified in the text, but it is stated that the levels of both BTK and its phosphorylated form (p-BTK) were “significantly lower” in the siRNA-treated group compared to the mock (saline) control group.
*TNF-α* (Tumor Necrosis Factor-alpha) [102]	LPS (10 mg/kg) intratracheal instillation	24 h before LPS instillation	Three doses of siRNA: 25 mg/kg, 50 mg/kg, 100 mg/kg of body weight	Exact numerical knockdowns (e.g., percentage reduction or absolute concentrations) are not presented in the text
*HDAC7* (Histone Deacetylase 7) [100]	endotracheal administration 30 × 10^6^ *Escherichia coli* (serotype 06:K2:H1, strain 19,138)	not specified	100 µg/100 µL per animal	Endotracheal administration of HDAC7-siRNA resulted in >40% suppression of HDAC7 protein synthesis in the lungs after 72 h.
Intravenously				
*LCN2* (Lipocalin-2) [99]	intraperitoneal administration of LPS 2 mg/kg	1 h after LPS injection	The authors of the study do not indicate the amount of plasmid with shRNA used.	The numerical values in percentages are not given in the text, but the graph shows that the reduction is more than 50% relative to the LPS + sh-NC group.
*NOX4* (NADPH oxidase 4) [103]	cecal ligation and puncture (CLP)	First siRNA Injection: Given 24 h before the CLP Injection Frequency: Administered once every 48 h.	1 mg/kg body weight.	Exact numerical knockdowns (e.g., percentage reduction or absolute concentrations) are not presented in the text
*TLR 4* (Toll-like receptor 4) [104]	intranasal instillation of LPS (20 μg/mouse in 100 μL PBS)	siRNA was administered immediately after LPS administration	100 mg/kg body weight.	Exact numerical knockdowns (e.g., percentage reduction or absolute concentrations) are not presented in the text

**Table 4:** Control groups and Main effect on the lung of siRNA used in in vivo to treat ALI/ARDS.

Target	Features of siRNA	Control Groups	Main Effect on the Lung	Reported Off-Target Effects/Toxicity
Intranasal				
*TIMP1* [96]	The siRNA selected during the study was used. Two siRNA sequences were compared, followed by their partial or complete modifications. Modifications containing 2′-O-methyl (2′-OMe) at nuclease-resistant sites were targeted to 204–224 nt or 500–520 nt of Timp1 mRNA. Animals were injected with siRNA in cationic liposomes 2 × 3-DOPE.	Two groups: siSCRm/2 × 3-DOPE scrambled siRNA + liposomes 2 × 3-DOPE only (Mock)—liposomes only	Histological Analysis siTimp1_2m resulted in a 2.5-fold reduction in the intensity of inflammatory changes in the lung tissue of mice exposed to LPS, while siSCR reduced this intensity by 1.4-fold. Administration of siTIMP1_2m resulted in a 1.8-fold reduction in the thickness of the interalveolar septa compared to control animals. BAL Fluid Analysis. Administration of siTIMP1_2m resulted in inhibition of leukocyte migration into BAL fluid, manifested by a 1.4- and 1.3-fold decrease in the proportion of neutrophils compared to control animals.	The authors of the study did not evaluate cytotoxic or off-target effects.
*BTK* [97]	siRNA from Invitrogen (Waltham, MA, USA) conjugated (T3-MAX conjugation kit, Bioo Scientific, Austin, TX, USA) with F(ab)2 fragments of anti-mouse neutrophil antibody (clone Ly-6G1A8) the main target is neutrophils	One group: LPS + control siRNA conjugated with F(ab)2 fragments and ICs (ALI/Cont siRNA group)	Histological Analysis Reduced lung inflammation/damage indices: alveolar hemorrhage, interstitial thickening, and the presence of alveolar exudates in mice treated with *BTK*-specific siRNA. After administration of siRNA to the *BTK*, a decrease was observed in Edema fluid to 0.163 Thickening of alveolar septa to 0.055 Inflammatory infiltration to 0.277, with values of 0.129, 0.055, and 0.20 6 in the saline control group.	The article provides specific data addressing the specificity of the intervention but does not report on systemic toxicity. The study demonstrates that the antibody-conjugated siRNA delivery system was highly specific to neutrophils. Btk expression was silenced in Gr-1^+^ neutrophils, but not in other cell types in the lung tissue.
*CCL2* [101]	The article does not indicate the origin of the siRNA. The siRNA was administered to animals as hybrid polymer-lipid nanoparticles (NPs) consisting of the cationic lipid-polymer compound 7C1 and PEG-lipid (C18-PEG5000) containing siCCL2.	One group: mock—animals that were not injected with the virus	*CCL2* silencing efficiency at 2 DPI: Significant reduction in CCL2 protein (ELISA, CBA) and mRNA (RT-qPCR) in lung homogenates. Immune cell modulation in lungs at 2 DPI—reduced infiltration of monocytes and neutrophils. No significant change in macrophage or dendritic cell counts. Lymphoid populations: decreased CD8^+^ T cells and increased NK^+^ cells in infected mice; NP-siCCL2 did not alter these populations. Viral load reduction: Significant decrease in lung PFU and viral RNA copies at 2 DPI and 4 DPI compared to untreated infected mice. Histopathological improvement: Reduced inflammatory infiltrate, alveolar thickening, and tissue damage at 2 DPI in treated mice. Systemic effects in spleen at 2 DPI: Restoration of CD3^+^ T cell numbers to mock levels in treated mice. No significant changes in myeloid cell populations in spleen. Cytokine expression at 2 DPI: Significant reduction in *IL-6* and *IFN-γ* mRNA in lungs of treated mice.	No significant systemic toxicity was reported. Histological evaluation of heart, kidney, spleen, and liver showed no morphological alterations, inflammation, or tissue damage. The nanoparticle formulation (7C1 + C18-PEG5000) was well-tolerated. No off-target immune effects or adverse events were noted in the study. The authors acknowledged that systemic CCL2 silencing could theoretically affect immune surveillance or tissue homeostasis, but no such effects were observed under the experimental conditions.
Intratracheal				
*BTK* [98]	The study used ready-made siRNA from Invitrogen. Animals were injected with naked siRNA. The main target is alveolar macrophages	Two groups: Mock group (M) was injected with an equal volume of saline and then CLP induced Sham group (S)—was given laparotomy and exploratory without CLP	Histological Analysis lung pathological score in the M group was significantly higher than that in the S group (5.47 ± 0.52, vs. 1.37 ± 0.16; *p* < 0.01), whereas the score in the Btk group mice, although higher than the S group (3.42 ± 0.33 vs. 1.37 ± 0.16; *p* < 0.01), was significantly lower than the M group (3.42 ± 0.33 vs. 5.47 ± 0.52; *p* < 0.01). The lung Apoptotic Index in the M group was significantly higher than in the Btk group (28.39 ± 2.74 vs. 19.78 ± 1.93; *p* < 0.01) The water content of lung tissues from the M group was significantly higher compared to the S group (82.04 ± 1.99% vs. 75.83 ± 1.16%; *p* < 0.01). The lung tissue water content in the *BTK* group was also significantly higher than in the S group (78.36 ± 2.16% vs. 75.83 ± 1.16%; *p* < 0.01), but significantly lower than in the M group (78.36 ± 2.16% vs. 82.04 ± 1.99%; *p* < 0.05).	The authors of the study did not evaluate cytotoxic or off-target effects.
*HDAC7* [100]	HDAC7-siRNA 21nt was designed by Rosetta algorithm prediction, targeting the HDAC7 gene 789 nucleotides downstream from the Refseq sequence.	One group—control siRNA (scrambled)	Histological Analysis significant reduction in neutrophil counts compared to the *E. coli* + control-siRNA group (*p* < 0.05). TNF-α in BAL fluid was significantly reduced by *HDAC7*-siRNA inhibition compared with control-siRNA (*p* < 0.05). Selective inhibition of HDAC7 increases survival by 3-fold.	None reported
*TNF-α* [102]	Commercially available siRNA by Heshen, Shanghai, China	One group: Sham group (S): Saline tail vein injection + LPS (10 mg/kg) 24 h later	Histological Analysis: - *Alveolar structure*: S group showed alveolar collapse, septal thickening, interstitial edema, and inflammatory infiltration. - Nanoparticle groups: Alveolar structure more intact, reduced congestion, and less inflammatory cell infiltration. - *Eosinophil counts* (per high-power field): - Nanoparticle groups: All significantly reduced eosinophil counts. But in 50 mg/kg group: significantly lower than 25 mg/kg and 100 mg/kg groups (*p* < 0.05). Protein Expression (Western Blot) - Nanoparticle treatment significantly reduced expression of TNF-α, Bcl-2, and Caspase-3 proteins. But TNF-α expression significantly lower in 50 mg/kg group than 25 mg/kg and 100 mg/kg groups (*p* < 0.05). Inflammatory Cytokine Levels (ELISA) All doses of siRNA-NP significantly reduced levels of TNF-α, IL-1β, and IL-6	None reported
Intravenous				
*LCN2* [99]	A commercially available shRNA delivery plasmid was used in this study. The shRNA sequence was not provided. The authors noted that the shRNA sequence was synthesized at Gene Pharma (Shanghai, China).	Two groups: Control—saline solution sh-NC group—administered control siRNA	*LCN2* depletion reduced lung wet/dry ratio indicating the alleviation of pulmonary edema by knockdown of LCN2. Histological Analysis: *LCN2* knockdown effectively counteracted the histological changes: numerous alveolar sacs, hemorrhage, alveolar wall edema and neutrophil infiltration.	No specific investigation or reporting of off-target effects or systemic toxicity was conducted in this study.
*NOX4* [103]	The control small interfering RNA (siRNA) (https://www.sciencedirect.com/topics/biochemistry-genetics-and-molecular-biology/small-interfering-rna, accessed on 1 January 2026) (Cat#: D-001210-01-50), specifically designed siRNA to NOX4 (https://www.sciencedirect.com/topics/biochemistry-genetics-and-molecular-biology/nox4, accessed on 1 January 2026) was obtained from Dharmacon (Chicago, IL, USA). For in vivo RNA interference, siRNA was prepared in cationic liposome-based Invivofectamine 3.0 Reagent (Cat#: IVF3005, Invitrogen, Grand Island, NY, USA).	Three groups: Sham—Abdominal incision only (no CLP) (N = 5) CLP + WT—CLP in wild-type mice (N = 14) CLP + Control siRNA—CLP + non-targeting siRNA (N = 15)	Survival Analysis (24 h post-CLP) Sham: 100% (n = 5) CLP + WT (Wild-type): 28.6% (n = 14) CLP + Control siRNA: 26.6% (n = 15) CLP + NOX4 siRNA: 52.9% (n = 17) (*p* < 0.01 vs. Control siRNA Wet/Dry Weight Ratio Sham: 4.5 ± 0.2 CLP + Control siRNA: 7.5 ± 0.3 (*p* < 0.01 vs. Sham) CLP + NOX4 siRNA: 5.0 ± 0.2 (*p* < 0.01 vs. CLP Control siRNA) Vascular Permeability (Evans Blue Index) Sham: 0.05 ± 0.01 CLP + Control siRNA: 0.25 ± 0.03 (*p* < 0.01 vs. Sham) CLP + NOX4 siRNA: 0.10 ± 0.02 (*p* < 0.01 vs. CLP Control siRNA) ROS Production (DHE Fluorescence Intensity) CLP increased DHE fluorescence by ~2.5-fold compared to Sham. NOX4 siRNA significantly reduced CLP-induced ROS (*p* < 0.01). Protein Expression (Western Blot) A. NOX Isoform Upregulation (2 h post-CLP) NOX4: 3.9-fold increase (*p* < 0.01 vs. 0 h) B. Phosphorylation of Signaling Proteins *p*-CaMKII (Thr286): Increased at 1 h post-CLP; NOX4 siRNA reduced phosphorylation by ~60% (*p* < 0.01). *p*-ERK1/2 & *p*-MLCK: Increased following CLP; NOX4 siRNA reduced phosphorylation by ~50–70% (*p* < 0.01). C. Tight Junction Proteins CLP reduced ZO-1 and Occludin expression by ~60–70%. NOX4 siRNA restored expression to near Sham levels (*p* < 0.01).	No specific investigation or reporting of off-target effects or systemic toxicity was conducted in this study.
*TLR 4* [104]	siRNA is commercially available (GenePharma, Shanghai, China)	One group: PBS control (LPS + PBS)	Histopathological Analysis: Neutrophil infiltration, hyperemia, edema, and capillary rupture in mice exposed to LPS were reduced in animals receiving Neutrophil-NP-TLR4. Immunohistochemistry TLR4, TNF-α, and IL-1β levels in the lungs were reduced by administration of Neutrophil-NP-TLR4. ELISA analysis Decreased concentrations of inflammatory cytokines, including TNF-α and IL-1β, in animals receiving Neutrophil-NP-TLR4. Western blot analysis LPS treatment increased TLR4 and NF-κB levels, while simultaneously decreasing AQP1 and AQP5 levels. After treatment with Neutrophil-NP-TLR4, TLR4 and NF-κB expression were significantly reduced, while AQP1 and AQP5 levels were increased (*p* < 0.001). Total BALF protein content The control group (PBS group) exhibited low BALF protein concentrations. LPS treatment significantly increased BALF protein concentrations, while Neutrophil-NP-TLR4 treatment significantly decreased total BALF protein content in ARDS mice, demonstrating that intratracheal administration of Neutrophil-NP-TLR4 improves alveolar capillary permeability in ARDS mice. Lung Wet-to-Dry Ratio LPS treatment significantly increased the wet-to-dry ratio, while Neutrophil-NP-TLR4 or NP-TLR4 treatment decreased this ratio compared to the LPS group. Specifically, the wet-to-dry ratio in the Neutrophil-NP-TLR4 group was very close to that in the PBS group.	Histopathological examination of the heart, liver, spleen, lungs, and kidneys after 15 days of treatment revealed no obvious pathological changes or signs of inflammation in the major organs of the Neutrophil-NP-TLR4 group compared with the PBS group. Blood biochemical parameters, including ALT, AST, white blood cells, red blood cells, and platelets, showed no significant difference between the PBS and Neutrophil-NP-TLR4 groups (*p* > 0.05), indicating no significant toxicity to normal cells.

Thus, among the target genes examined, the data suggest that *BTK* knockdown resulted in significant and, importantly, reproducible protective effects in two different ARDS models using different siRNA delivery systems [97,98]. In a study by Zhou et al., in an ARDS model reproduced by CLP, intratracheal administration of exposed *Btk* siRNA one hour prior to injury resulted in a marked reduction in inflammation, apoptosis, and pulmonary oedema. In a study by Krupa et al., which used a more sophisticated “two-hit” model (LPS priming followed by administration of an immune complex), *Btk* siRNA was conjugated with fragments of F(ab′)2 of an antineutrophil antibody (Ly-6G1A8) and administered intranasally after initial LPS exposure but prior to final reproduction of ARDS. This strategy has also resulted in a potent therapeutic effect characterized by increased apoptosis and efferocytosis of alveolar neutrophils. The reproducibility of the positive result, despite differences in the etiology of the models (polymicrobial sepsis versus immune-mediated inflammation) and delivery method (naked siRNA versus antibody-targeted conjugate), highlights the important role of *BTK* in the pathogenesis of ARDS and reinforces its value as a promising therapeutic target and suggests that the effect is not an artifact of a single experiment.

Although knockdown of other targets, such as *LCN2* and *CCL2*, also demonstrated profound effects, including reductions in oxidative stress, ferroptosis, viral load, and macrophage polarization, the data on *BTK* are supported by convergent data from independent research teams using different methodologies [99,101].

However, some studies suggest a more clinically relevant approach. For example, in the work of Krupa et al. on the introduction of *BTK* siRNA after the initial administration of LPS, interfering with the ongoing inflammatory process [97]. Similarly, the *CCL2* knockdown study involved the administration of siRNA 24 and 72 h after infection with the SARS-CoV-2 virus, which demonstrated efficacy in a model of already established disease [101]. These examples are more consistent with a true therapeutic intervention and provide more convincing evidence that siRNA can modulate an active pathological process.

In the presented studies, due to the lack of standardized presentation of data on knockdown efficiency (relative protein amount according to Western blot analysis or relative gene expression level according to PCR), the analysis of the correlation between knockdown efficiency and therapeutic outcome is complicated; however, based on the available data, a rough estimate of dose-dependent efficacy was made. In studies where the knockdown level was >50%, a pronounced clinical effect was observed, namely, in the work of Krupa et al., a knockdown efficiency of 92% correlated with a strong decrease in lung injury indices (alveolar hemorrhage, edema, infiltration) [97]; 50% knockdown of infection-induced *CCL2* mRNA led to a significant decrease in cell infiltration, viral load and improved histology; a decrease in the relative amount of HDAC7 protein > 40% led to a significant decrease in the number of neutrophils, TNF-α in BAL and a 3-fold increase in survival; 50% knockdown efficiency of *LCN2* resulted in comprehensive improvements including the control of ferroptosis. Thus, the knockdown level is a critical parameter for the effectiveness of siRNA therapy. Most targets require at least 40–50% suppression of expression to achieve a significant therapeutic effect. Analysis of the presented studies shows that the higher the knockdown level, the more pronounced the therapeutic effect. This seems logical, as interrupting key pathogenic cascades requires a significant reduction in the activity of the target protein.

Similarly to the lack of standardization in reporting knockdown efficiency, the studies reviewed used different types of controls:

1. Scrambled/non-targeted siRNA (sh-NC): This is the “gold standard” control. These oligonucleotides have a random sequence, not complementary to any known genome, but are similar in length, charge, and chemical structure to the active siRNA. They allow us to separate sequence-specific effects (gene targeting) from non-specific effects of the RNA molecule itself, activation of immune receptors (e.g., TLRs), or components of the delivery system. In the tables, this control was used for *TIMP1*, *HDAC7*, *NOX4*, *LCN2*, and *CCL2* (mock). This significantly increases the reliability of the data. The use of non-targeted siRNA against the background of ARDS induction in studies of *BTK* (Krupa et al.), *NOX4*, and *CCL2* demonstrates that the therapeutic effect is not a consequence of non-specific interference of the siRNA itself or the carrier in the pathological process [97].
2. Control with only the delivery system (liposomes, nanoparticles) or solvent (PBS, saline): This control (mock, PBS control) allows us to assess the impact of the administration procedure and the carriers themselves on disease development. For example, in the *TIMP1* knockdown study, there was a separate “2X3-DOPE only” group, which is appropriate, as liposomes themselves can exert an immunomodulatory effect. Similarly, in the TNF-α and TLR4 studies, PBS control was necessary to assess the underlying inflammation caused by LPS.
3. Sham group: Used in surgically induced models (CLP for *BTK* and *NOX4*). The Sham group undergoes all manipulations except the key damaging effect (the puncture itself and ligature), allowing for an assessment of the impact of surgical stress.

However, the study by Zhou et al. lacked a group with off-target siRNA, and comparisons were made only with the Mock (saline) and Sham groups [98]. This weakens the evidence base, as it does not completely exclude non-specific effects of naked siRNA administration. Also, many studies lack data on systemic toxicity or an assessment of off-target effects (with the exception of *CCL2* and *TLR4*, where this was done). Although scrambled siRNAs partially control for off-target effects, the use of transcriptome analysis methods to identify them would be ideal. Nevertheless, in most of the presented studies, the design of control groups is adequate and includes key elements (scrambled siRNAs, vehicle control), which increases the reliability of conclusions about the specificity of the studied siRNAs.

The presented studies demonstrate a wide range of approaches to siRNA delivery. Intranasal administration of siRNA offers the advantages of noninvasiveness, local delivery to the lungs, minimized systemic exposure, and the ability to target specific cells (neutrophils). However, it depends on the condition of the mucosa and mucociliary clearance and tends to be unevenly distributed. Intranasal administration in animals, analogous to nebulization in intubated patients, appears to be the most realistic route for clinical use of siRNA. Intratracheal administration, a relatively simple procedure in animals that is difficult to translate to humans, offers the significant advantage of direct delivery of siRNA to the lower respiratory tract. Use of the intravenous route is associated with a high probability of systemic exposure, the need for careful fine-tuning of the administration technique, and, most importantly, requires highly specific lung targeting systems (e.g., peptides, pulmonary endothelial antibodies). Without these, the risk of side effects is high.

It is also important to note that direct administration of siRNA into the respiratory tract (via instillation, inhalation, or nebulization) enables high local drug concentrations to be achieved directly in epithelial cells, alveolar macrophages, and other resident lung cells. In contrast, intravenous administration results in systemic siRNA distribution. Achieving therapeutic concentrations in the lungs then requires either very high doses or the use of complex targeting systems (e.g., those directed at the pulmonary endothelium) [50,51]. Thus, while local administration minimizes systemic exposure, it carries a significant risk of immunogenicity, as double-stranded RNA can be recognized by toll-like receptors (TLRs), particularly TLR3, TLR7, and TLR8, on the surface of pulmonary epithelium and alveolar macrophages. Unmodified or poorly modified siRNA, as well as drug contaminants, can activate these receptors. This triggers the local production of type I interferons (IFN-α/β) and proinflammatory cytokines (e.g., TNF-α, IL-6) directly at the site of inflammation [105]. In ARDS, this is tantamount to “adding fuel to the fire,” potentially exacerbating an existing cytokine storm. Such localized inflammation can increase pulmonary edema and cellular infiltration, which may mask the therapeutic effect or even worsen the patient’s condition. Notably, however, the risk of systemic off-target inflammation is low with this route, and severe manifestations like fever or systemic cytokine release are less likely than with intravenous administration. Proper siRNA design (using chemical modifications such as 2′-OMe or 2′-F) and biocompatible carriers can help mitigate these local side effects [44,96]. The key advantage remains the localization of the effect to the lungs, thereby avoiding systemic off-target effects in organs like the liver or kidneys. Conversely, systemic administration exposes siRNA and its carrier to a vast pool of immune cells throughout the body, including monocytes and macrophages in the liver and spleen, dendritic cells, B cells, and circulating neutrophils [106]. Activation via TLR7/8 in these cells can trigger a massive, systemic release of interferons and cytokines, leading to a condition resembling a “flu-like syndrome” (fever, myalgia, fatigue). For a critically ill ARDS patient on the verge of decompensation, this additional systemic inflammatory load can be particularly dangerous. Furthermore, intravenous administration is characterized by a pronounced “first-pass” effect through the liver, where most intravenously administered nanoparticles accumulate. This can lead to significant local immune activation in hepatic cells (hepatocytes, Kupffer cells), posing a potential risk of hepatotoxicity, as documented for some lipid nanoparticle (LNP) formulations [107]. A single, yet significant, advantage of systemic siRNA administration is its ability to target extrathoracic lesions in conditions like sepsis-induced ARDS. If the pathogenesis is triggered by a distant source (e.g., peritonitis), systemic administration could theoretically modulate inflammation at its origin. However, this approach demands absolute target specificity for the pathological process to avoid widespread adverse effects.

The strategy of targeting neutrophils with siRNA (via anti-Gr-1) in ARDS is promising and effective in clinical trials [97]. However, clinical translation requires addressing the immunogenicity of antibody conjugates, cost, and the search for even more specific markers of activated neutrophils in ARDS. Analysis of the presented studies also demonstrates the need for carriers: experience with naked siRNA demonstrated that even direct intratracheal administration is effective, but this is insufficient for clinical use [98]. The development of safe, effective, and stable nanocarriers such as lipid nanoparticles and polymer complexes is a prerequisite for moving to clinical trials. All studies used single siRNAs. Pooled siRNAs (a mixture targeting different regions of a single mRNA) could theoretically improve knockdown efficiency, but they complicate the regulatory process and increase the risk of off-target effects.

A serious problem with translating the results to the clinic, identified in the described papers, is the prophylactic or very early therapeutic administration of siRNAs in most experiments. For example, an anti-*Timp1* siRNA was administered 8 days prior to LPS-induced injury, an anti-*BTK* siRNA (Zhou et al.) was administered 1 h prior to CLP, and an anti-*HDAC7* siRNA was delivered 72 h prior to bacterial instillation [96,98,100]. This approach shows that suppressing these genes can prevent or markedly reduce the development of lung damage. However, it does not adequately model the clinical reality of ARDS, when treatment is initiated after the syndrome already has a vivid colic picture and the inflammatory cascade is fully active. This represents a major obstacle to clinical implementation, as the prophylactic efficacy of siRNA therapy cannot directly predict its efficacy in saving tissues from ongoing damage. The inflammatory environment of advanced ARDS, characterized by massive neutrophil infiltration, protease release, and vascular leakage, has the potential to disrupt exposed siRNA or interfere with the effectiveness of delivery systems, thereby reducing the therapeutic effect. From a methodological perspective, prophylactic administration of siRNA is rigorous and informative. It allows for clear proof of a causal relationship between the target gene and the pathogenesis of ARDS. If gene knockdown, performed before the onset of injury, prevents the development of the syndrome, this serves as direct evidence of the gene’s key role in initiating the cascade. This approach allows for the assessment of the maximum therapeutic potential of siRNA under idealized, controlled conditions, where the delivery system and siRNA molecules do not encounter barriers of established inflammation, and for the optimization of delivery and dosing parameters in models with a more predictable background. However, the translational interpretation of such data is extremely limited. The clinical reality of ARDS is that the patient is admitted to the intensive care unit with an already manifested, often rapidly progressing, syndrome. The physician is dealing not with the risk of development, but with an active, advanced pathological process. Consequently, the model of prophylactic siRNA administration does not adequately simulate the clinical scenario. Success in preventing injury does not guarantee success in reversing or halting it. This creates a significant gap between preclinical efficacy and clinical applicability, representing one of the key factors contributing to the high failure rate when moving from animal models to clinical trials (the so-called “valley of death” of translational medicine).

Therapeutic (post-traumatic) designs, where intervention occurs after the initiation of the pathological process, have greater clinical relevance. For example, in the study by Krupa et al., siRNA was administered 8 h after the first “hit” (LPS) but before the second (immune complexes) [97]. This model intervenes during the priming and progression phases of inflammation. In the study by Heloísa Athaydes Seabra Ferreira, siRNA to *CCL2* was administered 1 and 3 days after SARS-CoV-2 infection, that is, during the period of active viral replication and developing inflammation [101]. In the study by Xiaodong Wang, a plasmid containing shRNA to *LCN2* was administered 1 h after LPS, that is, during the incipient systemic inflammatory response [99]. These studies have immeasurably greater translational value. They demonstrate that siRNA therapy can modulate an already active inflammatory cascade, which is critically important, as in ARDS, many mediators are already released, cells are activated, and barriers are damaged. They also demonstrate the effectiveness of siRNA in developing ARDS, characterized by an abundance of proteases, nucleases, oxidative stress, and cellular debris, which can degrade siRNA and nanocarriers. Furthermore, these studies demonstrate that siRNA influences survival and damage resolution, not just its prevention.

The distinction between preventive and therapeutic designs for siRNA studies in ARDS is not simply a methodological nuance, but a fundamental question of translational relevance. While preventive studies prove the concept and identify key targets, they say little about actual clinical utility. The future of preclinical development of siRNA therapeutics for ARDS must be inextricably linked to strict adherence to the principle of clinical realism in experimental design. Only studies in which the intervention simulates a real-world clinical situation (treatment of an already established syndrome) will be able to generate reliable data justifying the transition to expensive and risky human clinical trials. Shifting the paradigm from “can this prevent disease?” to “can this cure active disease?” is essential for bridging the gap between promising laboratory results and the development of effective treatments for critically ill patients.

## 5. Conclusions

In summary, ARDS remains a devastating syndrome with a significant unmet need for effective pathogenetic treatments. The current therapeutic arsenal is limited to supportive measures, underscoring the critical importance of developing novel targeted strategies. Research into siRNA-based therapeutics offers a promising avenue by directly addressing the dysregulated gene expression that underpins ARDS pathology.

The successful application of siRNA in various animal models, from rodents to large pigs, demonstrates its potent efficacy in silencing key genes involved in inflammation, neutrophil infiltration, oxidative stress, and fibrosis. Knockdown of targets such as *TIMP1*, *BTK*, *LCN2*, *HDAC7*, *CCL2*, *NOX4*, *TNFα* and *TLR4* has consistently led to improved physiological parameters, reduced histological damage, and enhanced survival rates. These findings confirm the central role of these genes in ARDS pathogenesis and validate them as potential therapeutic targets.

However, the translation of these findings into clinical practice faces challenges. The choice of an appropriate animal model that accurately recapitulates human ARDS is complex, with each model having specific advantages and limitations regarding etiology, pathophysiology, and mortality. Furthermore, the development of safe and efficient delivery systems to target lung tissue remains a crucial area for future research.

Despite these challenges, the evidence presented strongly supports the continued investigation of siRNA therapy for ARDS. By enabling precise modulation of the disease’s molecular drivers, siRNA technology holds the potential to revolutionize ARDS treatment, shifting the paradigm from supportive care to targeted, mechanism-based intervention and ultimately improving outcomes for this critically ill patient population.

Looking ahead, the field is poised for transformation through several emerging technologies. Next-generation nanoparticle designs, such as those incorporating stimulus-responsive lipids or targeting ligands, promise to enhance the precision and efficiency of siRNA delivery to the lungs. Furthermore, the exploration of other RNA modalities, like antisense oligonucleotides (ASOs), could offer alternative strategies for modulating gene expression in ARDS. Finally, the use of ex vivo human models, particularly precision-cut lung slices (PCLS), is emerging as a powerful tool for high-throughput screening of therapeutic candidates, enabling rapid validation in a human-relevant system before advancing to complex in vivo studies [74].

Recent advances in RNA therapeutic technologies are poised to significantly impact the development of treatments for acute respiratory distress syndrome (ARDS) and other diseases. Novel nanoparticle designs, including lipid nanoparticles enriched with ionizable cationic lipids, biomimetic exosome–liposome hybrids, and mesoporous polydopamine nanoparticles coated with targeting moieties, have improved targeted delivery, stability, and immunogenicity profiles of RNA cargos such as siRNAs (small interfering RNAs). In addition to siRNAs, antisense oligonucleotides (ASOs) represent an expanding class of RNA modalities offering unique mechanisms of action for gene regulation, often with enhanced stability and cellular uptake due to chemical modifications. Complementing in vivo animal studies, the use of ex vivo human models such as precision-cut lung slices (PCLS) has emerged as a powerful platform for high-throughput screening of candidate therapeutics. PCLS retains the complex architecture and cellular heterogeneity of human lung tissue, enabling direct evaluation of inflammatory responses and gene expression changes relevant to ARDS pathophysiology while facilitating rapid screening of multiple drugs or RNA constructs prior to costly and lengthy in vivo validation. These innovations, supported by ongoing preclinical and mechanistic studies, collectively enhance the toolkit for developing effective, targeted RNA-based therapies for ARDS and other complex inflammatory lung diseases [74,96,101].

## Figures and Tables

**Table 2:** Main differences between siRNA and miRNA.

siRNA	miRNA
Origin [32,33]	
Generally exogenous (from outside the cell, e.g., introduced experimentally or viral RNA)	Endogenous (naturally encoded by the genome as non-coding RNA)
Structure [34,35]	
Double-stranded RNA, ~21–23 nucleotides with 2-nucleotide 3′ overhangs	Single-stranded from a hairpin precursor; mature miRNA ~19–25 nucleotides, forms imperfect duplex
Biogenesis [34,35]	
Derived from long double-stranded RNA processed by Dicer	Transcribed as primary miRNA, processed to pre-miRNA hairpin, then cleaved by Dicer
Target binding [32,36]	
Perfect or near-perfect complementarity to a single mRNA target	Partial complementarity, mainly binding 3′ UTR of multiple mRNAs
Number of targets [32]	
One specific mRNA target per siRNA	Multiple mRNA targets per miRNA, can regulate hundreds of genes
Mechanism of gene silencing [32]	
Cleaves target mRNA, leading to its degradation	Represses translation or destabilizes mRNA without direct cleavage
Function in the cell [32]	
Defense against viruses, transposons, or experimental gene silencing	Endogenous regulation of gene expression and fine-tuning of biological pathways

## Data Availability

No new data were created or analyzed in this study. Data sharing is not applicable to this article.
